# AntAlate—A Multi-Agent Autonomy Framework

**DOI:** 10.3389/frobt.2021.719496

**Published:** 2021-08-20

**Authors:** David Dovrat, Alfred M. Bruckstein

**Affiliations:** Multi-Agent Robotic Systems (MARS) Laboratory, Computer Science Department, Technion, Haifa, Israel

**Keywords:** unmanned aerial vehicles, multi agent robotic systems, software, framework, autonomy

## Abstract

AntAlate is a software framework for Unmanned Aerial Vehicle (UAV) autonomy, designed to streamline and facilitate the work of application developers, particularly in deployment of Multi-Agent Robotic Systems (MARS). We created AntAlate in order to bring our research in the field of multi-agent systems from theoretical results to both advanced simulations and to real-life demonstrations. Creating a framework capable of catering to MARS applications requires support for distributed, decentralized, control using local sensing, performed autonomously by groups of identical anonymous agents. Though mainly interested in the emergent behavior of the system as a whole, we focused on the single agent and created a framework suitable for a system of systems approach, while minimizing the hardware requirements of the single agent. Global observers or even a centralized control can be added on top of AntAlate, but the framework does not require a global actor to finalize an application. The same applies to a human in the loop, and fully autonomous UAV applications can be written in as straightforward a way as can semi-autonomous applications. In this paper we describe the AntAlate framework and demonstrate its utility and versatility.

## 1 Introduction

Unmanned mobile robotic platforms overcame barriers to reach an ever-growing user-base in recent years. From the military to the civilian domain, from graduate school laboratories to grade school classes, and from the highly specialized professional’s grasp to the enthusiastic hobbyist’s reach, applications of unmanned ground, surface, underwater and aerial vehicles have become widespread. The growing availability of low-power-high-performance mini-computers and micro-controllers, as well as the level of maturity and popularity reached by open-source software systems such as the Robot Operating System (ROS^1^), ArduPilot^2^, and Dronekit^3^, have made this prevalence possible. In their survey, Lim et al. (2012) explain and demonstrate how open-source UAV projects can empower the UAV application developer; the emergence of reliable software frameworks for UAV application development allows both professional and hobbyist developers to focus their efforts on the distinctive features of their own application while leaving the necessary yet onerous task of infrastructure development to the framework maintainers.

Though the topic of UAV design covers a large area, from frame configuration Eraslan et al. (2020) through flight controllers Kose and Oktay (2020) to software applications, we focus this discussion on the aspect of UAV software application framework design. Demeyer et al. (1997) describe frameworks as “semi-finished programs”; the applications being finalized by application developers that use the framework. The more functionality the framework offers, the more constraints it imposes on the future application developers. The framework designer must therefore resolve the conflicting tension between cross-context reuse and ease of adoption and adaptation. To balance the tension, Demeyer et al. offer guidelines to enhance three open system requirements: Interoperability, or the ability to run on various configurations; Distribution, or the ability to reliably run over a set of physically distributed nodes; and Extensibility, or the ability to finalize the application with added customization without having to change any of the framework’s internal modules. One of the primary dilemmas encountered by anyone trying to create a useful framework for multi-agent robotic systems (MARS) incorporating UAVs, is how much emphasis must be given to the particular UAV aspects of the framework; another dilemma is how to incorporate the swarm enabling multi-agent interaction mechanisms. Too much emphasis on UAV applications might leave the framework unfit for other platforms such as ground vehicles, while not giving the UAV platform enough consideration might leave the framework too high-level, requiring extensive tailoring from the ultimate application developer. Balancing the emphasis on swarm-enabling mechanisms is perhaps even more problematic, since any mechanism built into the framework limits the use of alternatives by future applications.

Chamanbaz et al. (2017), for instance, recently created the Marabunta^4^ framework built for enabling swarming capabilities to general purpose robotic systems, and demonstrated their framework’s capabilities in classic swarming scenarios for ground and surface vehicles. Preferring interoperability over distribution and extensibility to some extent, much of the implementation is left to the final application developer, and the framework’s synchronous calls to abstract functions from a single-threaded main loop per robot might become unfit for a UAV given a resource-demanding behavior. On the other hand, Preiss et al. (2017) described Crazyswarm^5^, a framework for indoor swarm applications using the Crazyflie^6^ platform, and the highly popular ROS middleware, used in conjunction with a global object tracker such as VICON^7^ for external feedback. While Crazyswarm applications perform most of their in-flight computation on-board the Crazyflie platform, a base station is required in order to calculate and broadcast pose estimates and is therefore an integral part of the Crazyswarm system architecture. Crazyswarm is therefore an example of a specialized framework willing to sacrifice generality for performance, as demonstrated in an impressive video featuring a swarm of 49 Crazyflies^8^. Arguably finding a middle ground between generality and specialization, Sanchez-Lopez et al. (2017) presented Aerostack^9^ - a framework designed as a set of components organized in a multi-layered model. Ultimate application developers can create their own application by selecting a set of components from the Aerostack component library and modifying or adding additional components as needed, as long as the developers adhere to the Aerostack conventions, thus satisfying the extensibility framework requirement. The interoperability and distribution requirements are achieved inherently by using ROS as underlying middleware for the single agent’s inter-process communication. Aerostack’s swarming capabilities are enabled by the framework’s social layer interface contracts, yet the mechanics of inter-agent communication is left for the application developer to finalize. A few examples of swarming solutions embedded into frameworks can be seen in the Voltron (Mottola et al., 2014), Buzz (Pinciroli and Beltrame, 2016), and CyPhyHouse^10^’s Koord (Ghosh et al., 2020) programming languages. Though varying in implementation details, the development framework provided by each of these languages allows the ultimate application developer to write an application from a swarm (or a sub-group of a swarm’s agents or super-group of sub-groups…) perspective with relative ease; this is done by including an underlying mechanism that propagates coordinating information between agents. Yet in applications where inter-agent communication is not required or even possible, these strengths become irrelevant, and with an increased number of agents the task of maintaining a distributed shared memory becomes a problem rather than a remedy.

For the past 20 years, our research team at the Technion MARS laboratory^11^ has been focusing on developing algorithms that address a variety of global tasks with swarms of simple mobile agents. Our paradigm defines agents as anonymous (i.e., not specifically addressable by an identifier), oblivious (have little or no memory), identical hardware platforms, that rely on locally acquired information provided by simple sensors such as local pheromone level detectors (Wagner et al., 1996; Wagner et al., 1999; Elor and Bruckstein, 2012a), proximity sensors (Gordon et al., 2008; Elor and Bruckstein, 2012b), or limited vision (Bellaiche and Bruckstein, 2017; Dovrat and Bruckstein, 2017) for their motion control decisions. Our work during these years led us to develop several types of local interaction-based motion rules for autonomous mobile agents in swarms deployed in various types of environments that achieve global tasks such as patrolling an area, gathering into a cohesive but flexible “cloud” of agents, coverage of regions for intruder detection, equitable distribution of workload, and path planning. See for example, the works of Wagner and Bruckstein (1997), Yanovski et al. (2003), Felner et al. (2006), Gordon et al. (2008), Osherovich et al. (2008), Elor and Bruckstein (2014), Elazar and Bruckstein (2016), Bellaiche and Bruckstein (2017), Dovrat and Bruckstein (2017), Altshuler et al. (2018), Manor and Bruckstein (2018), Amir and Bruckstein (2019), Barel et al. (2021), and Francos and Bruckstein (2021). We also addressed the issue of achieving guidance and steering of cohesive mobile agent swarms using some global “broadcast control” ideas, as presented in works by Segall and Bruckstein (2016), Dovrat and Bruckstein (2017), and Barel et al. (2018); where the broadcast signal is often assumed to be acquired by only a random set of the swarm’s agents. These ideas create a wealth of possibilities to deploy swarms of autonomous agents that can self-organize into cohesive, adaptive, and flexible-shaped constellations. These swarms can then be guided by a single user that communicates with the entire swarm *via* global broadcast signals based on observing the swarm’s location, but without having precise information on any particular agent of the swarm. It is easy to imagine the wealth of applications such a system can address, from site surveillance to disaster relief to space exploration. Yet the fundamental capabilities and limitations of swarms of such agents are rather difficult to analyze theoretically, so novel mathematical approaches are often needed to prove task accomplishment and termination, to evaluate the time span necessary to do the work, and to assess the effects of random or byzantine failures of agents. As examples of our team’s efforts we refer the reader to papers by Bruckstein et al. (1991), Bruckstein (1993), Altshuler and Bruckstein (2011), Oggier and Bruckstein (2012), Elor and Bruckstein (2012b), Segall and Bruckstein (2016), Barel et al. (2016), and Barel et al. (2021).

We created AntAlate^12^ to deploy swarms of agents that perform our algorithms in the real world. Considering its usefulness beyond implementing our algorithms, we hereby offer the framework to the multi-agent robotics R&D community, to facilitate the implementation of systems demonstrating various types of interesting swarming behaviors. AntAlate expresses our preference of UAV platforms, particularly copters, over others, since copters can emulate the behavior of wheeled and fixed-winged platforms to a greater extent than vice versa. AntAlate enforces an orderly execution of behaviors by means of a mission control (MC) module which interfaces with the high-level control (HLC) of the UAV and an operator module. Though we recommend a human at a control station as the operator, a centralized (or distributed) control server, or an on-board node for fully autonomous applications will also do. We included an operator station HTTP client (OSC) in the framework for all but the fully autonomous operator agents to use, and an operator station server for inter-agent and human interface as a complementary project^13^. By design, the swarming mechanism in AntAlate is amorphous, and can either emerge in a bottom-up fashion from the single agent’s behavior-set; be determined top-down by an operator; or any mixture of these approaches.

The remainder of this paper provides an in-depth description of AntAlate in Section 2, followed by a comparison of workflows when implementing the same algorithm to create ROS-based and AntAlate-based applications in Section 3, before concluding in Section 4.

## 2 Methods

A good framework provides the final application developers a convenient trade-off between the freedom to write their own application and the constraints imposed by having useful functionality they will find unnecessary to modify. Though any part of the code in an open-source project can be edited, the parts which the framework authors deem immutable can be considered as the framework’s *core*; developers are not required to alter these sections in order to write their own application. Hence, the framework core is generally where the benefits of using the framework present themselves. The framework’s extendable parts should be well defined by framework-contracts and mechanisms, such as abstract classes, that allow future developers to write modules that fit into the framework without significant overhead. The degrees of freedom the framework presents to the application developers can be thought of as a *design space*, where the framework contracts represent the axes, and different applications with different configurations can be represented by points in this space.

### 2.1 AntAlate Core

**FIGURE 1:**
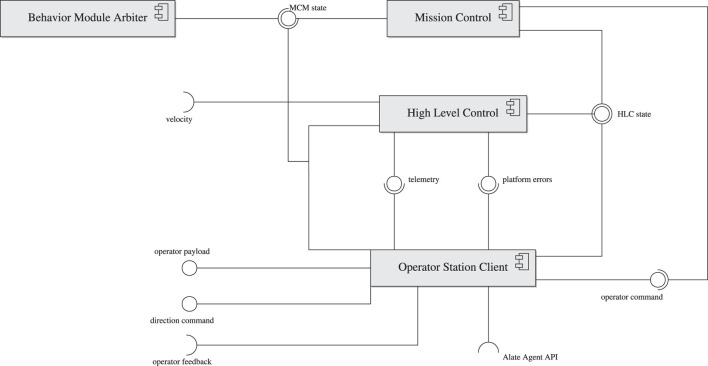
AntAlate core components and their interfaces.

The core functionality of AntAlate is to coordinate between an operator, a set of payloads, sensors and algorithms running onboard the UAV, and the UAV’s autopilot**.** Figure 1 shows a diagram of AntAlate’s core components. Each component is a NeMALA dispatcher^14^ node, communicating with other nodes by publishing messages to topics other interested nodes subscribe to. NeMALA^15^ is a set of supporting projects for AntAlate, with core components for dispatching, publishing, and handling messages, and tools^16^ to log and manage NeMALA dispatcher nodes and proxies. The dispatcher nodes are implemented in C++, utilizing Boost^17^, and ZeroMQ^18^, allowing nodes to communicate locally via inter-process communication, or TCP/IP if distributed over different computers. The ultimate application developers have control over the distribution of nodes, and can configure the method of communication between nodes by setting up NeMALA proxies catering their own project’s architecture and requirements, adding to the framework’s overall interoperability. AntAlate’s core components are the Mission Control (Section 2.1.1), High-Level Control (Section 2.1.2), Behavioral Module Arbiter (BMA; Section 2.1.3), and Operator Station Client (Section 2.1.4). Any future application requires only components of these four types, and some applications could do with less. Each of these components’ executables expect a NeMALA proxy name and a configuration file path as arguments when run from the command line (except the BMA, which is special in its requirement of its own node name instead of a proxy name). The configuration file contains the node number used for each node, as well as the proxy endpoints and topics used. Interoperability and distribution are therefore easily achieved by using one or many proxies described in one or many configuration files, without the need to alter code, recompile the application, or even edit configuration text-files, but only by calling the core executables with different arguments instead. The configuration file is also where behaviors, autopilots, and operator servers are specified, giving the ultimate application developers control over the AntAlate design space.

#### 2.1.1 Mission Control

**FIGURE 2:**
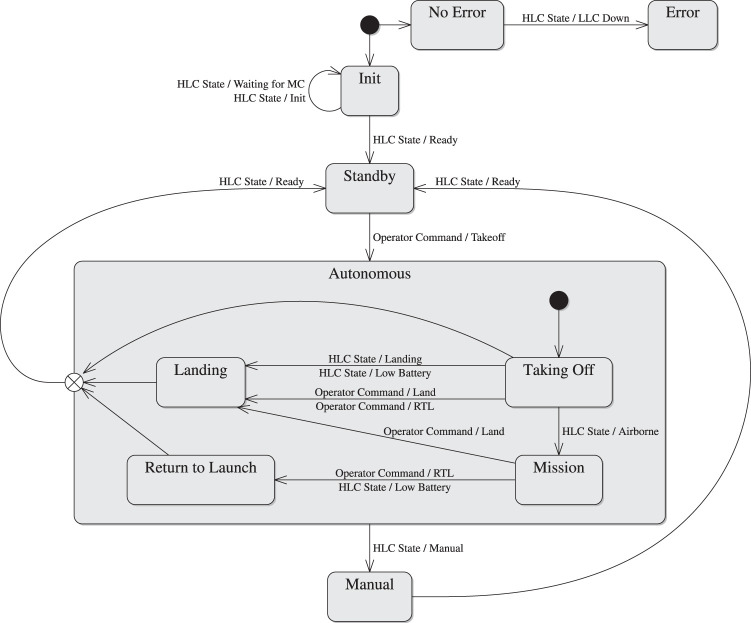
Mission control state machine.

The mission control component provides logic to coordinate all other AntAlate components by maintaining a state machine, illustrated in Figure 2, and publishing its state. The state machine’s inputs are operator commands and the HLC component’s state (see section 2.1.2); its output is the current mission state, which is provided as feedback to the operator, is used to initiate HLC processes, and perhaps most importantly from the framework point of view, governs the activation of sensors, payloads and algorithms by the Behavior Module Arbiter component (see section 2.1.3).

Upon initialization, the mission control synchronizes with the HLC component’s state machine and transitions itself to standby. An operator command to takeoff transitions the state machine to the autonomous states of taking off, performing a mission, returning to the launch site, and landing. A manual override initiated by a human pilot changes the HLC’s state to manual, causing a transition in the mission control state as well. The mission control can then return to standby only after the HLC returns to its ready state, meaning the UAV’s motors are disabled. If at any point the HLC reports that it is in its error state due to the autopilot shutting down, the mission state machine transitions to an unrecoverable error state.

#### 2.1.2 High-Level Control

**FIGURE 3:**
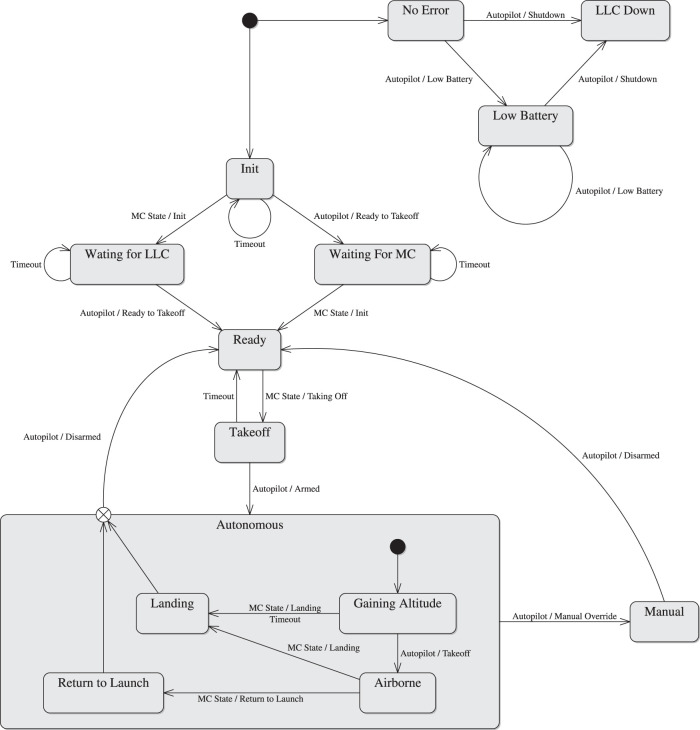
High-level control state machine.

The HLC component is an abstraction of the UAV platform. The HLC subscribes to the MC state topic and a velocity command topic, and publishes its state, telemetry and error data. The HLC state is decided by a state machine, illustrated in Figure 3, which coordinates the UAV autopilot abstraction with the MC state (see section 2.1.1).

When both autopilot and MC are up, the HLC enters its ready state. The HLC responds to a MC transition to its taking off state by making an attempt to arm the UAV’s motors while transitioning to the takeoff state. Failure to arm the motors brings the HLC state back to ready; success brings about a transition to gaining altitude, where the autopilot attempts to gain enough altitude to be considered airborne before running out of time and being forced to land by a transition to the landing state. While airborne, an MC transition to its landing or return to launch states causes the HLC to follow suit. A manual override is possible in any of the autonomous states. After landing and disarming the motors, the HLC returns to ready mode from either manual or autonomous states. If at any point the HLC loses communication with its autopilot, the state machine transitions to the unrecoverable state LLC down to inform the MC and Operator that the vehicle is about to crash. Low battery transitions the state machine to a low battery state.

#### 2.1.3 Behavior Module Arbiter

The Behavior Module Arbiter activates behavior-sets according to a state topic it subscribes to. Multiple BMAs can be cascaded such that the root BMA subscribes to the MC state, and publishes its own arbitrary state for other BMAs to subscribe to. The BMA gives AntAlate an added degree of freedom in the distribution of behaviors over separate nodes, as well as being key to AntAlate’s extensibility by activating plugin behaviors (see section 2.2.1). The configuration file given as an argument to the BMA executable tells the node which behavior set to run, and which proxies to subscribe to.

#### 2.1.4 Operator Station Client

**FIGURE 4:**
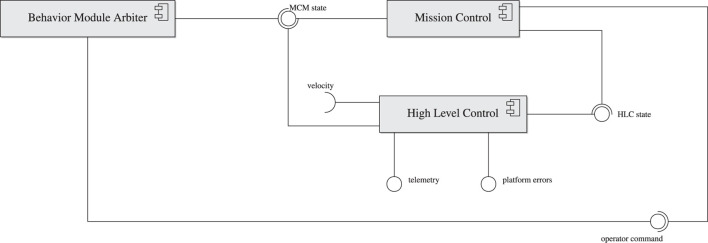
AntAlate minimal deployment.

AntAlate requires an operator node to publish commands such as takeoff or land to the operator command topic. For example, a minimal operator node could be a BMA which publishes a takeoff command to the operator command topic every time the MC enters its standby state, as can be seen in Figure 4. Yet, in order to facilitate inter-agent, human-agent or human-swarm communication, we added an Operator Station Client that serves as an anonymous client to a server *via* HTTP, as can be seen in Figure 1. The OSC accepts tokens from the server, so anonymous protocols can stay anonymous, but any protocol requiring agents to be labeled is also supported. The OSC subscribes to the MC and HLC topics and forwards the messages to the server. The server replies with an operator command or a direction command if one was recently given, and the OSC publishes the commands received to their appropriate topics. In addition, outgoing operator payload and incoming feedback topics are left for the final application developers to use as they see fit. The server IP address and port are specified in the application’s configuration file, which the OSC reads at runtime while setting up the node.

### 2.2 AntAlate Design Space

Swarming protocols generally differ not only in the way their agents behave, but also in the way their agents sense the environment and communicate among themselves or with an external operator. UAV systems usually differ in type of flying platform and the autopilot providing low-level control over the flying platform. The AntAlate design space therefore is composed of three major axes: Behavior (Section 2.2.1), Autopilot (Section 2.2.2), and Communication (Section 2.2.3), with Sensing split in implementation between these major axes.

#### 2.2.1 Behaviors

We created AntAlate in order to easily implement and test new swarming behaviors on UAVs; during the design process, though, we found that there are other uses for the behavior mechanism other than swarming protocols, such as single-UAV autonomy, payload management, and sensing. Ultimately, the behavior mechanism can be used as a building block to create almost any type of UAV application.

**FIGURE 5:**
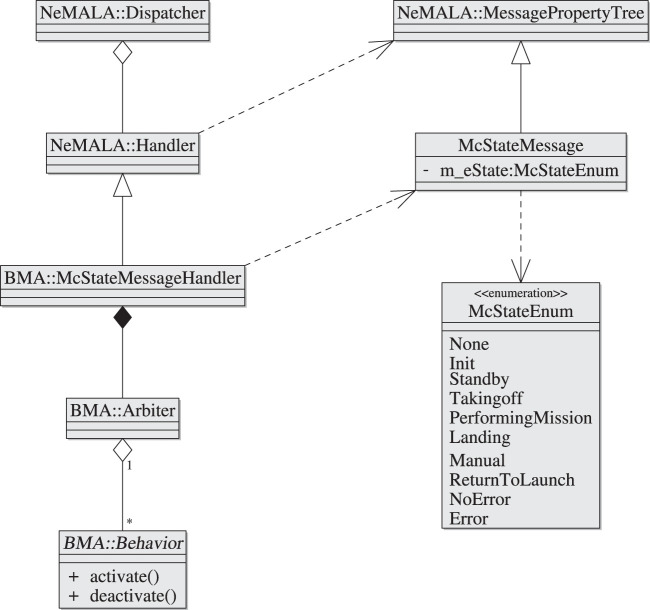
AntAlate’s Behavior Module Arbiter class diagram. The behavior abstract class is a framework contract.

Figure 5 shows the reusable design in the form of a class diagram; a BMA node is a NeMALA dispatcher that has a McStateMessageHandler which handles incoming McStateMessage type messages that arrive *via* a topic the BMA registers to. These messages encapsulate an instance of an enumeration type that represents a mission-state. The handler has an Arbiter which maps mission states with concrete behavior classes, and activates or deactivates its behaviors accordingly whenever a message containing a recognized state is received.

Using a plugin mechanism to populate arbiters with behaviors, the BMA generically controls the activation of behaviors while leaving the behavior specifics to future programmers. Behavior interaction is made easy by adding topics to publish and subscribe to, and BMAs can be cascaded by having behaviors publish mission-state messages to designated topics other than that of the original MC. To create a new behavior, one must create a shared library containing a class derived from the behavior abstract class and a concrete factory class to which the BMA delegates the construction and configuration of the behavior, along with its integration into the BMA node.

To add a behavior to an application, all that is required is that the application’s configuration file includes an entry for that behavior, including which mission states activate and deactivate the behavior, the plugin library name, and to which topics the behavior subscribes and publishes to. No need to recompile the framework to change the configuration, even when adding new behaviors.

#### 2.2.2 Autopilots

**FIGURE 6:**
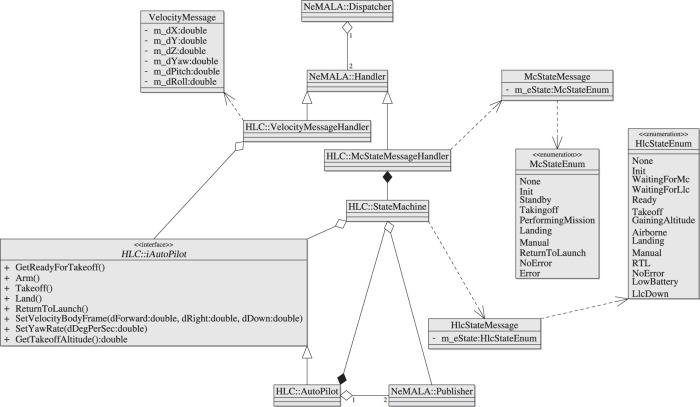
AntAlate’s High Level Control class diagram. The iAutoPilot interface is a framework contract, the concrete autopilot is a Python-C++ bridge.

Autopilots, in this discussion’s scope, are the hardware/software components that serve as an intermediate between the actual UAV platform and AntAlate logic, including behaviors, operators, and the mission control. The HLC and its autopilot class diagram are shown in Figure 6. AntAlate’s modular design allows the extension through inheritance of the autopilot interface class to fit to a specific UAV API. The HLC’s concrete autopilot class implements a Python-C++ bridge to facilitate the integration of python based APIs such as Dronekit^19^ and Tello^20^. By using a bridge we can extend the concrete autopilot class without recompiling the AntAlate code-base; additional autopilot APIs can be covered by the same concrete autopilot class by adding Python implementations and updating a factory python script. Ultimate application developers can then choose which autopilot API to use by altering the application’s configuration file.

#### 2.2.3 Communication

The OSC (Section 2.1.4) provides some degree of freedom to the design space by means of the operator payload and feedback topics, yet imposes a specific HTTP request and expects the server’s reply to be formatted in a specific way. We deliberately excluded a server from the framework to emphasize that the server we produced^21^ only represents one example out of infinite possibilities. We encourage future application developers to use the OSC without alteration, and to write their own server, tailored for their application’s behaviors, users, and use cases.

Though we find it useful, the OSC is not the only extra-agent channel of communication allowed in AntAlate, and is indeed not even the only form of operator that falls into the constraints of the framework. Other operators can be implemented using the behavior mechanism, and other communication methods can be added as behaviors as well. In this context, inter-agent communication can be regarded as an AntAlate behavior, with a BMA node using any type of hardware/software communication stack, protocol, etc. NeMALA proxies, topics and messages can be used as well as general building blocks for future applications.

## 3 Results

**FIGURE 7:**
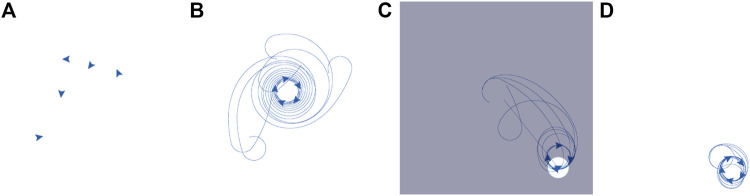
Our algorithm implemented with NetLogo. Five agents are initially dispersed at random on the plane **(A)**. The agents gather to a rotating regular pentagon **(B)**. Dragging one of the agents to the bottom left corner of the arena, the rest of the agents follow and ultimately form a rotating square about the “dragged leader” **(C)**. “Releasing” the leader, it returns to the swarm, and again a rotating pentagon forms, at the new location **(D)**.

We used AntAlate to implement a swarming algorithm we previously described and implemented using ROS^22^ and TurtleBot2^23^ platforms (Dovrat and Bruckstein, 2017). In this section, we will present our workflow using AntAlate and compare it with our ROS workflow, which may be familiar to most readers. We usually start our workflow with NetLogo^24^ (Wilensky, 1999) simulations to help refine our algorithms and to quickly make observations to base our theoretical hypotheses on. Figure 7 shows a NetLogo simulation of our algorithm^25^ where a swarm of five agents are manipulated by the user taking over (drag-and-dropping) one of them.

Once satisfied with the results, we can choose a suitable mobile platform and design our application. Our swarming algorithm is fairly simple: every agent either detects other agents in its field of view and turns gently to one direction, or does not detect other agents and turns sharply to the same direction. In other words, the algorithm’s input is a boolean valued true if other agents are detected or false otherwise, while the algorithm’s output is a real value representing angular velocity, which switches between two predefined values according to the input.

**FIGURE 8:**
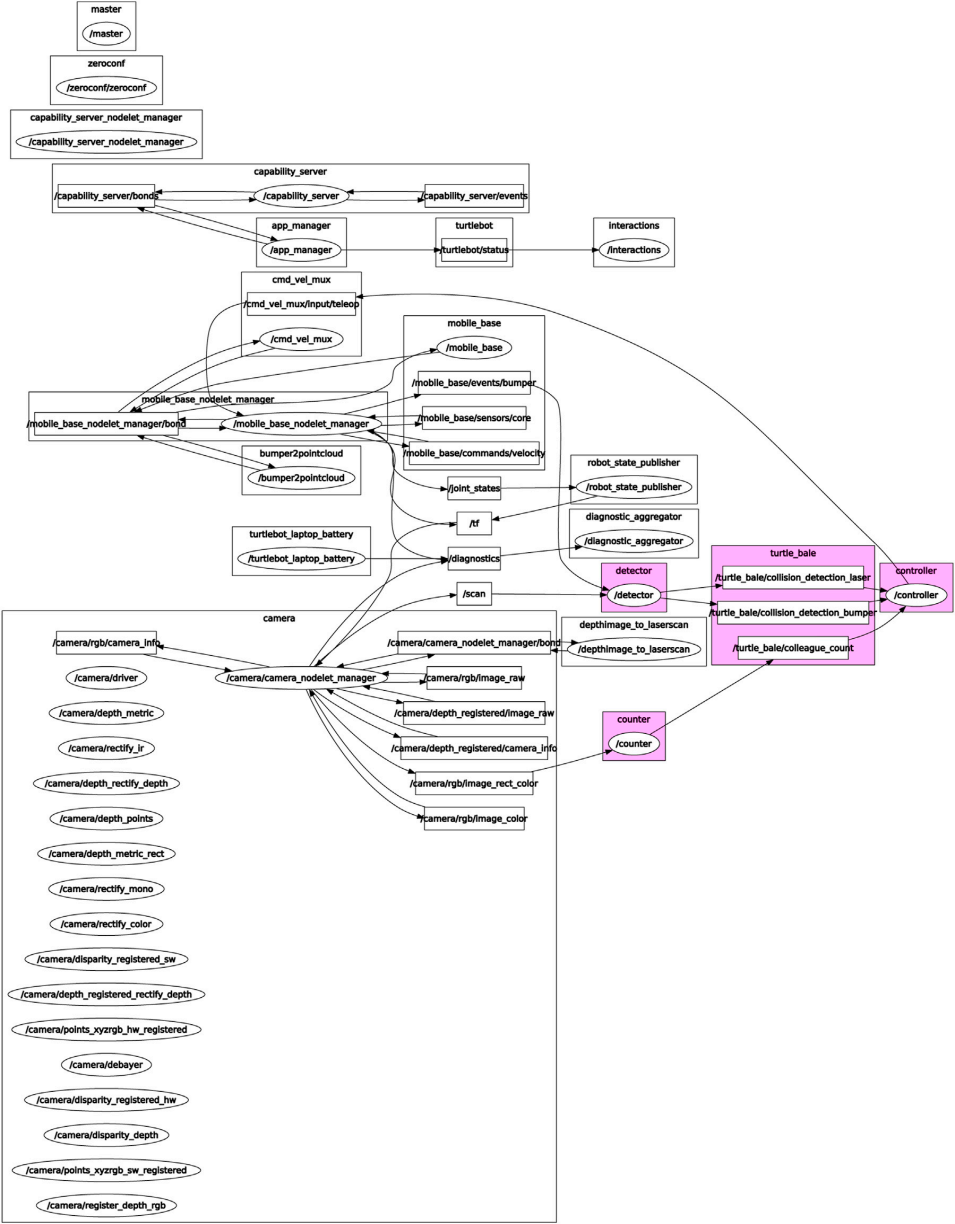
Our Turtlebot application’s ROS graph. The application topics and nodes written by us are highlighted, while the rest of the graph was made available to us by the ROS community.

**FIGURE 9:**
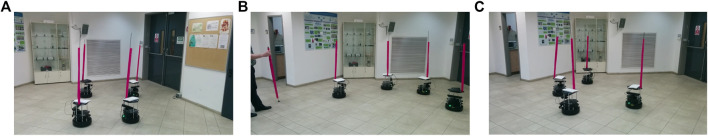
Our algorithm implemented with TurtleBots and ROS. The agents gather to a formation resembling a rotating square **(A)**. The agents follow a human leader **(B)**. When the leader goes away, the agents eventually return to their previous formation at a new location **(C)**.

The TurtleBot2 platform is perfectly suited for handling this algorithm, and the next step is to see which interfaces fit our algorithm’s needs. Figure 8 shows the ROS graph of our application^26^. To detect other agents, we created a counter node which counts the number of magenta colored poles in an image frame and publishes the result. Figure 9, taken from this a short video^27^, shows our robots fitted with clearly visible colored rods, following a hand-held rod which acts as a leader, similar to the simulated behavior shown in Figure 7. Capitalizing on the TurtleBot2 capabilities, we added a detector node which reports if the agent has bumped into something or if its laser scanner has detected an obstacle nearby. The controller node translates the detected rod count to “false if zero, true otherwise,” and executes our algorithm along with an overriding obstacle avoidance procedure if necessary. The controller then publishes a message with the correct forward velocity and turning rate to the relevant topic the robot’s velocity command multiplexer subscribes to.

**FIGURE 10:**
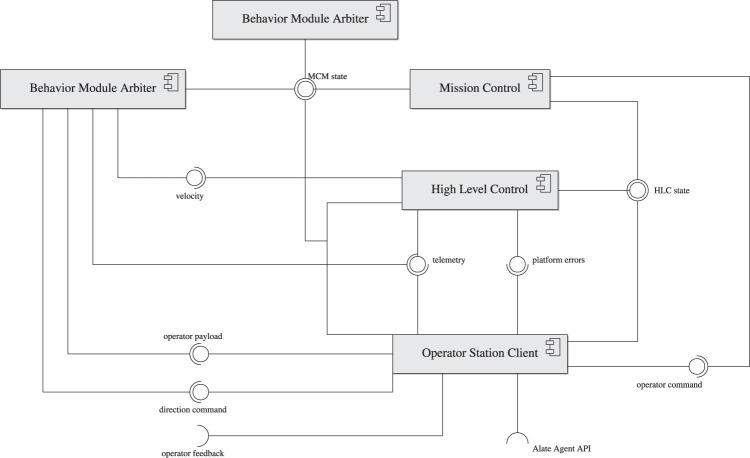
AntAlate template application deployment.

Our process using AntAlate was similar, and we included the swarm algorithm, as well as a video capture behavior, as a template application in the AntAlate code repository. Figure 10 shows the template application’s deployment diagram, where each of the two behaviors gets its own BMA node. The swarm algorithm’s BMA subscribes to the direction command, telemetry, and operator payload topics from which it derives the direction the operator wants the swarm to move towards, the azimuth the agent is moving towards, and the existence of peer agents in a sector in front of the agent, respectively. The algorithm BMA’s output is a velocity command which the HLC subscribes to, and which incorporates the operator’s direction command with the swarm algorithm’s output. The video capture behavior uses the command line tool ffmpeg^28^, which requires separate installation on the target machine, to capture video from a device specified in the application’s configuration file; it neither subscribes nor publishes to any topic. Both BMAs subscribe to the MC state topic in order to activate their behaviors when appropriate.

**FIGURE 11:**
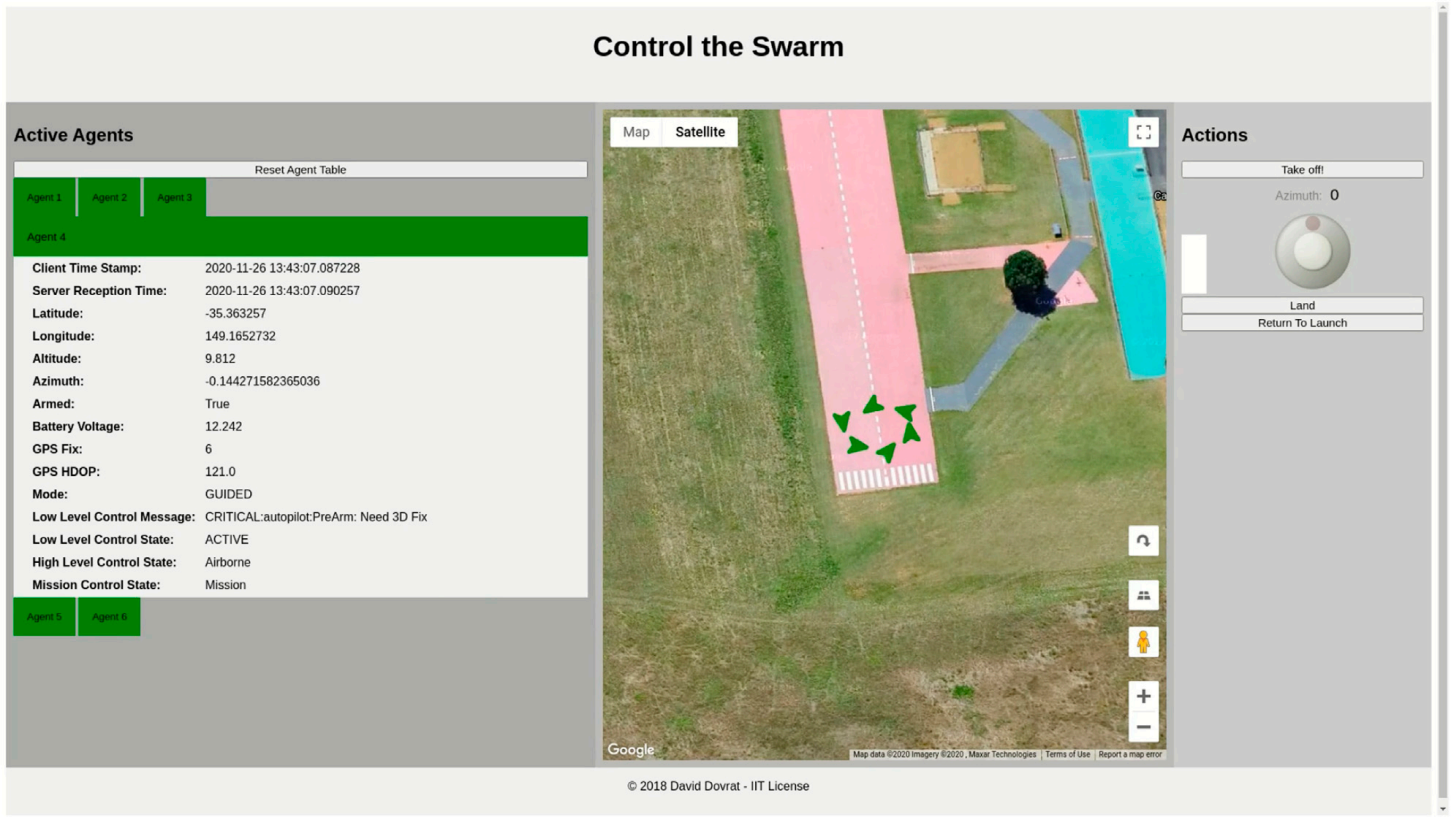
AntAlate template application demonstrated using ArduCopter’s Software in The Loop (SITL) simulator. Screen capture from the AntAlate Server Graphical User Interface (GUI).

**FIGURE 12:**
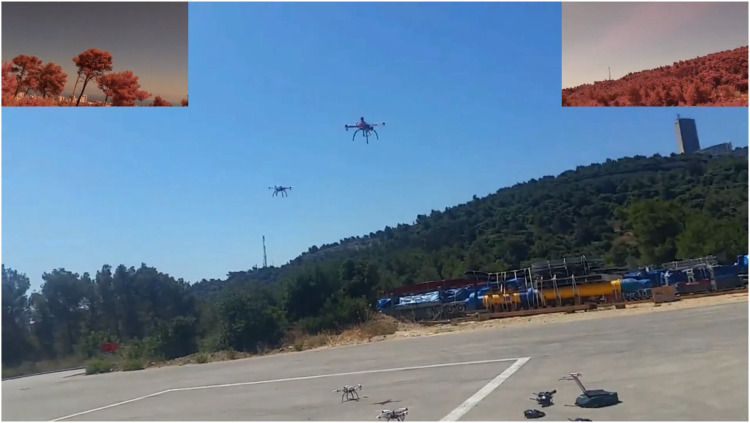
AntAlate template application demonstrated with 470 mm quadcopters. Frames captured by the video capture behavior are presented in the upper corners.

**FIGURE 13:**
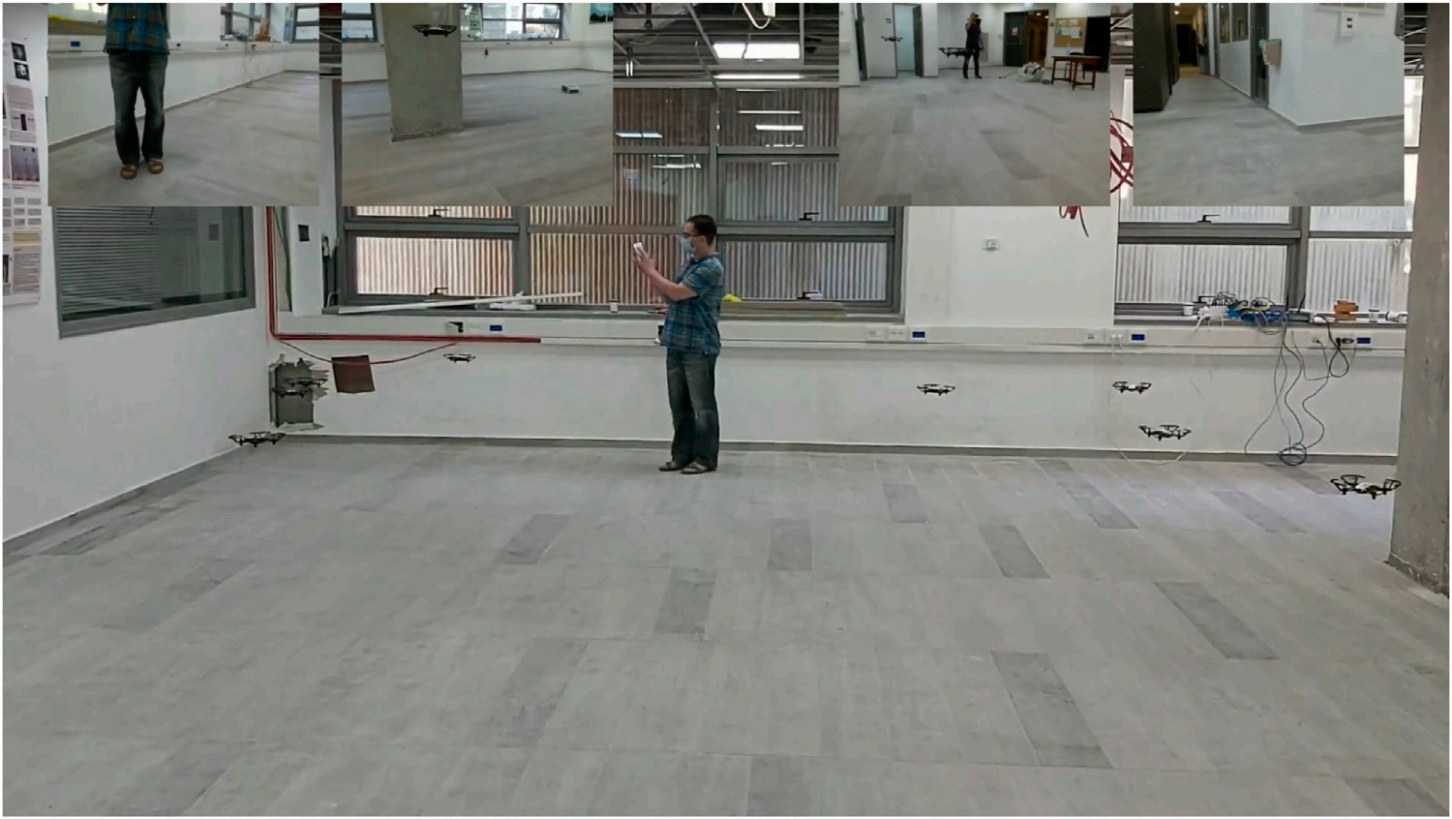
AntAlate template application demonstrated with Tello platforms. The upper part of the figure shows frames from the video capture behavior of four of the agents.

AntAlate’s modular and configurable design makes it fit for deployment using containers; we created docker^29^ images for each AntAlate component type (MC, HLC, OSC, BMA), for linux/arm and linux/amd64 architectures, as well as two BMA images with pre-built behaviors, one for the swarm algorithm and one for the video capture behavior. We made these images publicly available on Docker Hub^30^. With these docker images we deployed the same code to three different configurations: A simulation that uses an external SITL ArduCopter^31^ simulator as an autopilot and communicates with it *via* TCP; a 470 mm UAV frame with a pixhawk^32^ autopilot and a Raspberry Pi^33^ onboard that runs AntAlate and communicates with the pixhawk through a mavlink^34^ serial connection; and a Tello^35^ with a companion Raspberry Pi that communicates with the autopilot *via* wifi using the Tello API. Figures 11–13 are taken from a video^36^ featuring these configurations. From a design-space point of view, the first two configurations use the same Dronekit^37^ autopilot, though on a different computer architecture, and the last two use the same computer architecture but with two different autopilots. The simulation, having no video devices, doesn’t run a video capture BMA. We chose to run the template application’s behaviors on separate nodes, but one BMA node would have sufficed.

The template application’s algorithm requires the detection of other agents as an input, yet the simulated agents have no camera or sensing device capable of detecting other agents, so we compensated for the missing ability by using a tailored server along with the OSC. The OSC’s HTTP post request includes the HLC state which in turn includes the agent’s location and orientation. When a new agent posts its state for the first time, our server^38^ assigns an index to it for further updates. The server keeps a data base, and each time an agent updates the server with its state, the server records the state and solves the inverse geodesic problem using Geographiclib^39^ to answer whether another agent is in the sector in front of the updating agent in the HTTP reply’s payload field. The detection range and field of view are server parameters. The OSC parses the HTTP reply to publish to the AntAlate operator command and direction command topics if necessary, and forwards the payload to the AntAlate operator payload topic. The BMA running the swarm algorithm subscribes to the payload topic and receives the server-calculated response as its required detection input. Had there been a peer detecting sensor, a BMA encapsulating that sensor would have published to some peer-detection topic, the swarming algorithm would have subscribed to that topic instead of the operator payload topic, and the server’s database would have been unessential to the application. Which topic the component subscribes to is detailed in the configuration file given as input to AntAlate components.

Though the resulting applications are very different, the first being a ground robot that can avoid and handle bumping are one item and not two obstacles, and the second an aerial robot that accepts broadcast signals, the workflow was almost identical: come up with an algorithm, build a process that uses available interfaces to encapsulate the algorithm, refine the resulting process to take advantage of available assets and compensate for missing assets, simulate, test, and deploy.

Looking at the amount of bytes in manually written files as an indicator of human effort, both workflows are comparable. The ROS application weighs 13,191 bytes not including the counter and collision detection nodes, and 23,638 bytes including them. the AntAlate application’s behavior plugin code and configuration files weigh 18,118 bytes. This example showcases the power of a good framework: a few hundred lines of custom application code utilize thousands of lines of framework code. With AntAlate, we use the same nodes over and over; we code a new behavior once and configure it, as well as combine it with other behaviors, to form new applications without going over the entire design process for each added behavior. The application design is mostly implemented in the framework up until the point of concrete behaviors, which are left for the custom application developers to program and deploy according to their application’s requirements and constraints.

## 4 Discussion

This paper introduces and describes AntAlate, a software framework we created for the future development of UAV MARS applications. AntAlate is a manifestation of our multi-agent systems paradigm; we chose a system of systems approach and focused on the single agent, while avoiding constraints on the swarming mechanism, expressing our preference for local over global sensing and communication, anonymity and obliviousness over distributed and shared memory, decentralized autonomy over centralized control. We incorporated an operator entity as a means of inter-agent and agent-human communication in an effort to keep the framework useful for MARS applications outside the scope of our paradigm notwithstanding.

We identified the UAV-MARS design-space and enforced framework contracts that promote maintainable future extensions. We employed proxies with configurable endpoints to enable the physical distribution of AntAlate nodes, and created a modular, interoperable, architecture which allows future developers to code once and deploy the same code on many different platforms and simulators, as we demonstrated with an example application.

Future framework enhancements include general types of operators that bridge between underlying frameworks and communication modals, in addition to the existing HTTP client. An example of such an operator could be a ROS node operator that exposes and forwards the AntAlate topics to ROS topics and vice versa, allowing the AntAlate agent to integrate into ROS applications. In addition, the development of some useful behaviors, such as peer recognition, obstacle avoidance, and simultaneous localization and mapping, could prove useful for developers interested in using these behaviors as building blocks in their own application without having to re-implement the wheel. Integrating a wider range of autopilots into the framework is another development priority, with the Crazyflie API^40^ at the top of the autopilot backlog.

We hope the multi-agent robotics community will find AntAlate useful, and that AntAlate becomes the framework of choice for easy implementation of many interesting swarming behaviors, as well as an instrument for future collaborations and discussions.

## Data Availability

A code repository containing the software framework described in this study is available at https://gitlab.com/nemala/alate.

## Footnotes

1
https://www.ros.org/

2
http://ardupilot.org/

3
https://dronekit.io/

4
https://github.com/david-mateo/marabunta

5
https://github.com/USC-ACTLab/crazyswarm

6
https://www.bitcraze.io/crazyflie-2-1

7
https://www.vicon.com

8
https://www.youtube.com/watch?v=D0CrjoYDt9w

9
https://github.com/Vision4UAV/Aerostack

10
https://cyphyhouse.github.io/index.html

11
https://mars.cs.technion.ac.il/

12
https://gitlab.com/nemala/alate

13
https://gitlab.com/nemala/operator-station

14
https://gitlab.com/nemala/core

15
https://gitlab.com/nemala

16
https://gitlab.com/nemala/tools

17
https://www.boost.org/

18
https://zeromq.org/

19
https://dronekit.io/

20
https://github.com/dji-sdk/Tello-Python

21
https://gitlab.com/nemala/operator-station

22
https://www.ros.org/

23
https://www.turtlebot.com/turtlebot2/

24
http://ccl.northwestern.edu/netlogo/

25
http://ccl.northwestern.edu/netlogo/models/community/dovrat2017

26
https://gitlab.com/dave.dovrat/turtle_bale

27
https://www.youtube.com/watch?v=OA4ri3X4izw

28
https://ffmpeg.org/

29
https://www.docker.com/

30
https://hub.docker.com/u/nemala

31
https://ardupilot.org/dev/docs/sitl-simulator-software-in-the-loop.html

32
https://pixhawk.org/

33
https://www.raspberrypi.org/

34
https://mavlink.io/

35
https://www.ryzerobotics.com/tello

36
https://youtu.be/i9kctIlLkTgshort

37
https://dronekit.io/

38
https://gitlab.com/nemala/operator-station

39
https://geographiclib.sourceforge.io/html/python/index.html

40
https://github.com/bitcraze/crazyflie-lib-python
